# Inoculation of *Bacillus velezensis* Bv-116 and its bio-organic fertilizer serve as an environmental friendly biocontrol strategy against cucumber *Fusarium* wilt

**DOI:** 10.3389/fpls.2024.1467265

**Published:** 2024-10-15

**Authors:** Haolong Li, Shanshan Zhao, Xueying Zhang, Fengyuan Yang, Changsong Feng, Yuhang Huang, Xiaoxue Tang, Pintian Sun, Yanping Wang

**Affiliations:** ^1^ Henan Key Laboratory of Ion Beam Bio-Engineering, School of Agricultural Science, Zhengzhou University, Zhengzhou, China; ^2^ Henan Province Engineering Research Center of Traditional Chinese Medicine Transformation for Chronic Diseases, Zhengzhou Hospital of Traditional Chinese Medicine, Zhengzhou, China; ^3^ Basic Medical Research Center, Academy of Medical Sciences, Zhengzhou University, Zhengzhou, China; ^4^ Institute of Animal Husbandry and Veterinary Science, Henan Academy of Agricultural Sciences, Zhengzhou, China

**Keywords:** cucumber, *Fusarium* wilt, *Bacillus velezensis*, bio-organic fertilizer, Biocontrol mechanism

## Abstract

The aim of this study was to evaluate the effects of *Bacillus velezensis* Bv-116 and its bio-organic fertilizer on the control of cucumber *Fusarium* wilt caused by *Fusarium oxysporum* f. sp. *Cucumerinum* (FOC), the promotion of growth of cucumber seedlings, and the soil microbial community. *B. velezensis* Bv-116 exhibited an inhibition rate of 84.93% against FOC, as well as broad-spectrum inhibitory activities against other soil-borne plant pathogenic fungi. Fermentation products of *B. velezensis* Bv-116 destroyed the cell structure of FOC and inhibited the growth of FOC mycelium. These products were identified as volatile antimicrobial gases, proteases and cellulases. In the greenhouse pot experiment, both *B. velezensis* Bv-116 and its bio-organic fertilizer exhibited significant promoting effects on cucumber growth, and a significant reduction in the incidence and disease severity index of cucumber wilt (*p* < 0.05). Analysis of the microbial community structure of cucumber rhizosphere soil revealed that inoculation of *B. velezensis* Bv-116 and its bio-organic fertilizer increased the abundance of genera with biocontrol capabilities against plant pathogens. In addition, inoculation of the bio-organic fertilizer reversed the excessive proliferation of *Fusarium* and Acidobacteria. Our results suggest the potential of inoculating *B. velezensis* Bv-116 and its bio-organic fertilizer as an environmentally friendly biocontrol strategy against cucumber wilt.

## 1 Introduction

Cucumber (*Cucumis sativus* L.) is a common, popular and economically important vegetable crop. Cucumber wilt caused by *Fusarium oxysporum f.* sp. *Cucumerinum* (FOC) is one of the most damaging soil-borne fungal diseases, which is widely distributed, greatly restricting the yield and quality of cucumber, and causing great economic losses worldwide (Bartholomew et al., 2022).

Biological control is considered to be an eco-friendly, safe and long-lasting effective method to control the incidence of diseases, and has been hailed as the most promising control pathway at present (Sarrocco, 2023). Recent studies have shown that biological control of wilt using biocontrol strain antagonists and their bio-organic fertilizer is a promising strategy. For instance, The volatile compounds produced by *Streptomyces albulus* NJZJSA2 isolated from soil samples exhibited a significant inhibitory effect on the germination of spores of *Fusarium oxysporum* (Wu et al., 2015). *B. velezensis* AP-3 protects tomato against *Fusarium* wilt and promotes growth of tomato plants under salt stress (Medeiros and Bettiol, 2021). Addition of *Trichoderma harzianum* SQR-T037 bio-organic fertilizer to both nursery and transplanted soils diversified the microbial community in the continuous soil, resulting in effective control of cucumber wilt (Chen et al., 2012).

Meanwhile, studies have revealed that the addition of biocontrol bacteria in organic fertilizer can regulate the soil microecological environment, enhance soil fertility, while providing abundant nutrients for plants, promote plant growth and development, thereby improving plant disease resistance (Tahir et al., 2017). With the increase of kitchen waste year by year, it brings great harm to the environment. Adding biocontrol bacteria to kitchen waste and fermenting to prepare bio-organic fertilizer, thus greening kitchen waste and reducing environmental pollution, will be a valuable research direction in the future.

In view of the harmful effects of FOC-induced cucumber *Fusarium* wilt on the yield and quality of cucumber, the aim of this study was to screen for an antagonistic bacterial strain with significant inhibitory effect against FOC and to explore an environmentally friendly biocontrol strategy against cucumber wilt. In this study, *Bacillus velezensis* BV-116 was screened and identified as a biocontrol strain with antagonistic activity against plant pathogenic fungi such as FOC. The potential antimicrobial mechanism of BV-116 was investigated. Then, it was added to kitchen waste fermentation to make bio-organic fertilizer with potential control of cucumber *Fusarium* wilt. Through greenhouse pot experiments, the study explored the growth-promoting and disease prevention effects of BV-116 and its bio-organic fertilizer, as well as their impact on the structure of rhizospheric soil microbial communities.

## 2 Materials and methods

### 2.1 Microbial strains and culture conditions

The bacterial strains used in the study for screening and fungal strains (*Trichoderma longibrachiatum*, *Aspergillus niger*, *Aspergillus oryzae*, *Aspergillus flavus* and *Fusarium graminearum*) were obtained from Henan Key Laboratory of Ion Beam Bio-Engineering, Zhengzhou University. *Fusarium oxysporum* f. sp. *Cucumerinum* strain Race-4 was obtained from College of Plant Protection, Henan Agricultural University. All of the above strains grown on potato dextrose agar (PDA) medium were stored in a 4°C refrigerator.

### 2.2 Antifungal activity assay

The antagonistic bacteria were screened by the plate confrontation method. Cultured colony solution was sucked up and evenly spread on the surface of Nutrient Agar (NA) plates, which were then inverted and incubated at 37°C for 24h. With a punch, respectively take 6.5 mm in diameter of pathogen mycelial plug (from colonies edge), the candidate strains medium plug and sterile NA medium plug. The pathogen mycelial plug was placed in the center of the PDA plate, and two candidate strain medium plugs and two sterile NA medium plugs were placed opposite each other at the four corners 15 mm away from the plug. The sterile NA medium plug was used as the control. This process was repeated three times. The plates were incubated at 28°C for 7 days, and the diameter of the pathogenic fungus was measured to calculate the inhibition rate (Zhang et al., 2019).

### 2.3 Identification of antagonistic strain

#### 2.3.1 Morphological observation

The antagonist strains were inoculated onto NA plates by Streak plate method and incubated at 37°C for 24h. Colony characteristics were observed and Gram staining was performed, and the individual characteristics and staining results were observed by laser confocal scanning microscope (ZEISS LSM780, Germany) and optical microscope (Olympus CX31, Japan), respectively.

#### 2.3.2 The 16S rDNA analysis

The 16S rDNA gene sequencing of the selected strain was performed as described by Li et al. (2015).

### 2.4 Identification of antagonistic component of *B. velezensis* Bv-116

#### 2.4.1 Effect of *B. velezensis* Bv-116 fermentation supernatant on FOC

The FOC was inoculated into potato dextrose broth (PDB) at 28°C and 180 rpm for 4 days, and then in PBS solution to reach a pathogen spore concentration of 1 × 10^7^ cfu/mL. *B. velezensis* Bv-116 was inoculated in NB medium and cultured at 37°C and 180 rpm for 48 h. The supernatant was then collected and filtered through a 0.22 μm microporous sterile filtration membrane to obtain the sterile fermentation supernatant.

The effect of *B. velezensis* Bv-116 fermentation supernatant on spore germination rate and inhibition rate were determined using the concave slide method (Yang et al., 2018). The effect of supernatant on the morphology of FOC mycelium was determined following the procedure described by Chen et al. (2014). The effect of supernatant on FOC cell membrane permeability was determined following the procedure described by Zhou et al. (2018).

#### 2.4.2 *Bacillus velezensis* Bv-116 antagonistic gas detection

Nutrient Agar culture medium was poured into the lid of a sterile culture dish, cooled and then 100 µL of *B. velezensis* Bv-116 fermentation supernatant was added and spread evenly. PDA culture medium was added to the culture dish, and a 6.5 mm diameter mycelium plug containing the pathogenic fungus (FOC) was placed in the center. The dish was covered with a lid and sealed with parafilm before being inverted and placed in the incubator. The side with the inoculated pathogenic fungi facing downwards and the side coated with Bv-116 facing upwards. The dish was incubated at a constant temperature of 28°C, and the growth of the pathogenic fungus was observed. NA culture medium without fermentation supernatant in the lid of the culture dish was used as a control.

#### 2.4.3 *Bacillus velezensis* Bv-116 extracellular enzyme activity assay

The *B. velezensis* Bv-116 was inoculated separately on skim milk agar medium (Zhai et al., 2021), colloidal chitin medium (Zhai et al., 2021), and Poria cocos powder medium (Park et al., 2012). The cultures were then incubated at a constant temperature of 37°C for 24 h, and the formation of clear zones was observed. The objective was to determine whether *B. velezensis* Bv-116 exhibited protease, chitinase, and β-1,3-glucanase activities, respectively.

*B. velezensis* Bv-116 was inoculated onto sodium carboxymethylcellulose solid medium and incubated at a constant temperature of 37°C for 24 h. Cellulase activity of *B. velezensis* Bv-116 was determined using Congo red staining (Meddeb-Mouelhi et al., 2014).

### 2.5 Antimicrobial stability test of *B. velezensis* Bv-116 fermentation supernatant

The supernatant of *B. velezensis* Bv-116 fermentation was taken in 15 portions, with each portion containing 5mL. Among them, 5 portions were subjected to continuous heating at 40, 60, 80, 100, and 120°C for 15 minutes. Another 5 portions were placed at a distance of 25 cm from a UV lamp and irradiated for 1, 3, 5, 7, and 9 h, respectively. The remaining 5 portions were adjusted to pH 3.0, 5.0, 7.0, 9.0, and 11.0 using 1 mol/L HCl and NaOH, and left at room temperature for 12h before readjusting the pH back to its initial value of 7.2. The inhibitory activity of the aforementioned fermentation supernatant was evaluated using the double-layer agar plate method (Feng et al., 2021).

### 2.6 Pot experiment

#### 2.6.1 Preparation of FOC spore suspension and *B. velezensis* Bv-116 cell suspension

The *B. velezensis* Bv-116 strain was inoculated into Luria-Bertani (LB) medium and cultured at 37°C with shaking at 180 rpm for 24 h. Subsequently, the cells were harvested by centrifugation, resuspended in PBS solution, and adjusted to a concentration of 1 × 10^8^ cfu/mL. Resuspend the spore suspension of FOC in PBS solution and adjust its concentration to 1 × 10^6^ cfu/mL.

#### 2.6.2 Preparation of nutrient soil and bio-organic fertilizer

Nutrient soil was prepared by mixing peat soil (Pinnacle Top Group, Denmark) and vermiculite (Baitouji, Shijiazhuang Red Grass Trading Co., Ltd.) at a mass ratio of 2:1. The kitchen waste used in the experiment was collected from Dongyuan Restaurant, Jiahui Fresh Food Supermarket, and Wanbang International Seafood Wholesale Market, in Zhengzhou, Henan Province. The main components were mixtures of decayed fruits, discarded vegetable leaves, and slaughter wastes of chickens, ducks, and fishes (blood, intestines, scales, gills, and so on). Then, *B. velezensis* Bv-116 bacterial suspension was inoculated at a concentration of 1×10^8^ cfu/g dry weight, stirred and mixed, and then fermented for 90 days at room temperature (approximately 24°C) to produce bio-organic fertilizer.

#### 2.6.3 Cucumber seedlings and culture

Cucumber seeds (cultivar Fuyang No. 2) were soaked in 0.1% HgCl_2_ solution for 1 min, washed and air-dried, and then incubating at 4°C for 10 h before constant dark incubation at 28°C. When the radicle grew to about 0.5 -1 cm, the seeds were sown in seedling trays containing sterilized nutrient soil (5×10 holes, Hunan Hezhiyuan Seed Industry Co., Ltd.), and placed in the greenhouse at 28°C, with a light intensity of 10,000 Lux, relative humidity of 60%, and a light cycle of L:D=16:8 for cultivation.

#### 2.6.4 Determination of the growth promoting effect of *B. velezensis* Bv-116 and its bio-organic fertilizer

When the cucumber seedlings in the seedling trays reached the three-leaf stage, they were transplanted into 12cm diameter pots, with one cucumber seedling per pot. 3 groups were set up for the experiment, and the design was as follows: CK, pots were filled with 3000 g of nutrient soil; T1, pots were filled with 3000 g of nutrient soil containing 3×10^10^cfu *B. velezensis* Bv-116.; T2, pots were filled with 2700 g of nutrient soil and 300 g of bio-organic fertilizers (containing 3 × 10^10^ cfu of *B. velezensis* Bv-116 at the start of fermentation) and mixed homogeneously.

Adjustment with sterile water made the moisture content the same in each treatment group, and each treatment group was divided into three groups of 15 seedlings each. All seedlings were cultivated under greenhouse conditions at 28°C, light intensity of 10,000 Lux, relative humidity of 60%, and photoperiod L:D=16:8 for 20 d. Plant height, stem thickness, leaf fresh weight, leaf dry weight, root fresh weight, root dry weight, and chlorophyll content were determined.

#### 2.6.5 Determination of disease prevention effect of *B. velezensis* Bv-116 and its bio-organic fertilizer

When the cucumber seedlings in the seedling trays reached the three-leaf stage, they were transplanted into 12cm diameter pots, with one cucumber seedling per pot. 4 groups were set up for the experiment, and the design was as follows:CK1, the pots were filled with 3000 g of nutrient soil, and the roots of cucumber seedlings were soaked in sterile water for 20min and then transplanted into the pots; CK2, pots were filled with 3000 g of nutrient soil, and the roots of cucumber seedlings were soaked in FOC spore suspension for 20min and then transplanted into pots; T1, pots were filled 3000 g of nutrient soil containing 3×10^10^cfu *B. velezensis* Bv-116, and the roots of cucumber seedlings were transplanted into pots after soaking in FOC spore suspension for 20min; T2, 2700 g of nutrient soil and 300 g of bio-organic fertilizers (containing 3 x 10^10^ cfu of *B. velezensis* Bv-116 at the start of fermentation) were added to the pots and mixed homogeneously, and the roots of cucumber seedlings were soaked in the FOC spore suspension for 20min and then transplanted into the pots.

Cucumber seedlings were continued to be cultured under the above conditions for 20 d and investigated and recorded separately. The disease incidence (DI), disease severity index (DSI) and control efficacy (CE) were calculated according to the method of Huang et al. (2020).

#### 2.6.6 Analysis of cucumber rhizosphere soil microbial communities

After the cucumber seedlings continued to be cultured for 20 d under the above conditions, the rhizosphere soil of cucumber plants from each of the four treatment groups was taken and the soil samples were mixed homogeneously between the replicate treatment groups, 10 g of the soil samples were mixed with 90mL of sterile phosphate buffer and incubated for 1h at 180 rpm, then filtered through four layers of sterile gauze, and the filtrate was centrifuged at 4°C for 15min at 8000 rpm to collect the precipitate samples, and DNA of the fungi and bacteria in the samples was extracted separately by the use of a kit (Omega Fungal and bacterial DNA were extracted from the samples using kits (Omega Biotek, Norcross, GA, USA), DNA concentration was determined using a NanoDrop ND-2000 spectrophotometer, and DNA quality was assessed using 1% agarose gel electrophoresis. PCR amplification of the ITS1 region of the fungus was performed using forward primer F (Illumina adapter sequence 1 + CTTGGTCATTTAGAGGAAGTAA) and reverse primer R (Illumina adapter sequence 2 + GCTGCGTTCTTCATCGATGC). The bacterial 16S rDNA V3- V4 region was PCR amplified using the forward primer Primer F (Illumina adapter sequence 1+ CCTACGGGGNGGCWGCAG) and the reverse primer Primer R (Illumina adapter sequence 2+ GACTACHVGGGTATCTAATCC). PCR products were extracted using a 2% agarose gel, and each sample was purified by adding an equal volume of AgencourtAMpure XP Nucleic Acid Purification Magnetic Beads to obtain the raw library of the samples. The PCR products were purified, quantified, and then sequenced by the Miseq platform (Shanghai Tianhao Biotech Co. Ltd., China). All raw reads were checked using FLASH2 (version 1.2.11), and low-quality sequences with quality scores below 20 were filtered out according to the QIIME quality control process (version 1.9.1). Samples were analyzed for Alpha diversity, Beta diversity and microbial community composition.

### 2.7 Statistics and analyses

The data of this study were collated and analyzed using Microsoft Excel 2010 with Origin 2018 software, data such as spore germination inhibition and diameter of the Inhibition circle were analyzed by general linear model using IBM SPSS 21.0, and differences between treatments were evaluated using the Duncan test, and the height of cucumber seedlings was measured using Image-J software. Alpha diversity was calculated using Mothur software (version 1.9.1), and Beta diversity was analyzed using the R package (version 2.15.3). Tukey’s multiple comparisons test was applied to determine statistical differences between means, which were considered significant when *p* < 0.05.

## 3 Results

### 3.1 screening and identification of antagonistic bacteria

**Figure 1:**
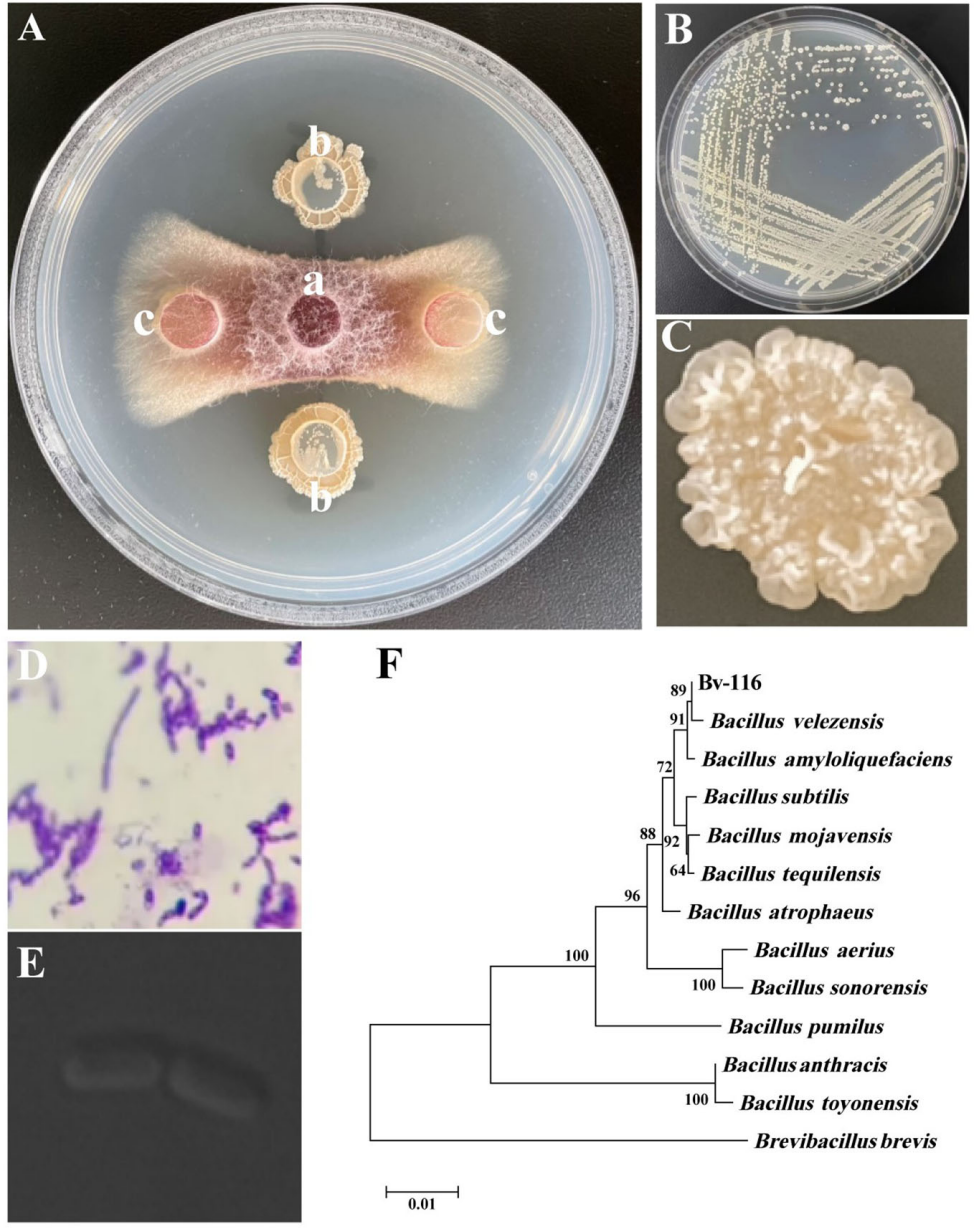
Screening and Identification of FOC antagonistic strain Bv-116. **(A)** Antagonistic effect of Bv-116 against FOC, in **(A)**, a is the mycelial plug of FOC, b is the bacteria-carrying medium block of Bv-116, and c is the sterile NA medium block; **(B, C)** Colony morphology of Bv-116; **(D)** result of Bv-116 Gram staining; **(E)** Laser confocal scanning microscopy observations of Bv-116; **(F)** Phylogenetic trees based on 16S rDNA.

Ten strains exhibiting significant antagonistic activity were screened from 565 bacterial strains maintained in the laboratory, using FOC as an indicator fungus (**Supplementary Table S1**). Among them, strain Bv-116 showed the highest inhibitory effect on the pathogen (**Figure 1A**) with 84.93% inhibition. The single colony of this strain milky white, opaque, with a dry, wrinkled raised surface and irregular edges (**Figures 1B, C**), Gram staining yielded positive results (**Figure 1D**), and laser confocal scanning microscopy revealed the strain to be rod-shaped (**Figure 1E**). Morphological observations, combined with 16S rDNA gene BLAST results and phylogenetic tree analysis (**Figure 1F**), conclusively identified the strain as *Bacillus velezensis*. *B. velezensis* Bv-116 exhibited inhibition rates exceeding 85% against all five tested fungi, including *Fusarium graminearum* (**Supplementary Figure S1**, **Supplementary Table S2**).

### 3.2 Preliminary investigation on the inhibitory effect of *B. velezensis* Bv-116 on FOC and its potential mechanism

**Figure 2:**
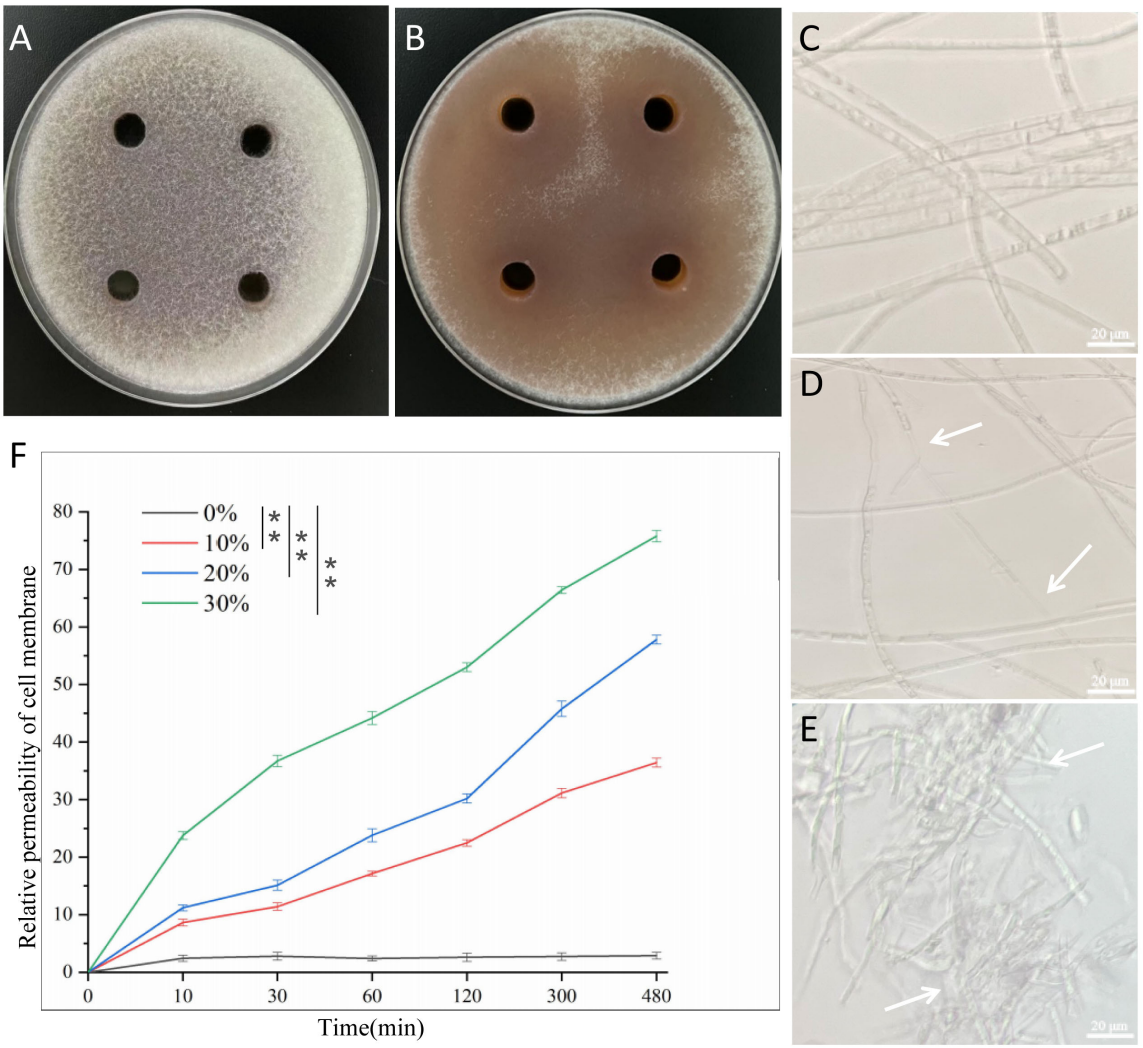
Effect of *B*. *velezensis* Bv-116 fermentation supernatant on spore germination, mycelial morphology and cell membrane permeability of FOC. **(A, B)** Germination of FOC spores in the presence or absence of *B*. *velezensis* Bv-116 fermentation supernatant; The growth status of FOC mycelium without **(C)** and with **(D, E)** the presence of Bv-116; **(F)** Effect of *B*. *velezensis* Bv-116 fermentation supernatant on the relative permeability of FOC cell membrane. In the same picture, ** indicate significant differences at the *p* < 0.01 level.

The plates treated with fermentation supernatant of *B. velezensis* Bv-116 showed obvious inhibition circles compared with the control (**Figures 2A, B**), When the fermentation supernatant concentration was 10%, 20% and 30%, the inhibition rates of FOC spore germination were 37.99%, 64.34% and 86.93% (**Supplementary Figure S2**), respectively. The results showed that the fermentation supernatant of *B. velezensis* Bv-116 had a significant inhibitory effect on spore germination of cucumber *Fusarium* wilt, and the inhibition rate gradually increased with the increase of fermentation supernatant concentration.

As indicated by the white arrow in the figure, under the action of *B. velezensis* Bv-116, the mycelial growth of FOC exhibited deformities, the increase of irregular mycelium, the mycelium entangled with each other, the mycelium adhered to form groups, the partial dissolution of mycelium and the protoplasm in mycelium was unevenly distributed, and the contents were lost (**Figures 2C-E**). When the inhibited mycelia were re-inoculated into PDB medium, their growth activity was significantly reduced, and their growth rate was notably slower (**Supplementary Figure S3**).

The results of relative permeability of the cell membrane of FOC with time (**Figure 2F**) showed that following treatment with *B. velezensis* Bv-116 fermentation supernatant at different concentrations for varying durations, the relative membrane permeability of the cell membrane of FOC increased over time and with the concentration of fermentation supernatant, significantly exceeding that of the blank control group. After treatment with 30% fermentation supernatant for 480 min, the relative permeability of the FOC cell membrane reached 75.77%.

**Figure 3:**
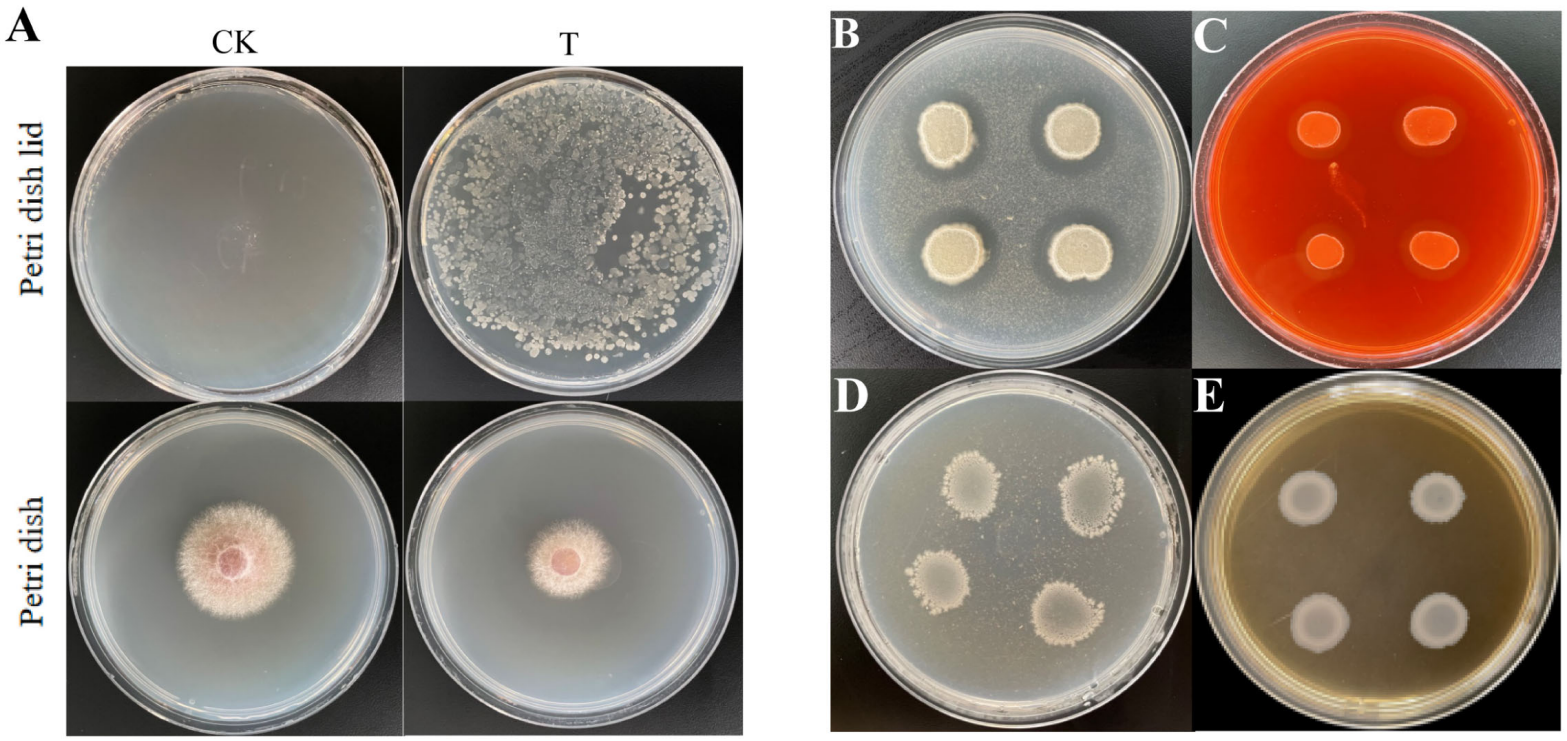
Determination of antagonistic gas and extracellular enzyme activity of Bv-116. **(A)** The petri dish lid on the left and the petri dish are the control group without *B. velezensis* Bv-116 inoculation (CK), and the treatment group inoculated with *B. velezensis* Bv-116 is on the right (T); **(B)** Detection of protease; **(C)** Detection of cellulase; **(D)** Detection of chitinase; **(E)** Detection of β-1,3-glucanase.

Volatile gases produced by *B. velezensis* Bv-116 significantly inhibited the growth of FOC in comparison with the normal growing FOC in the control group (**Figure 3A**).

The colonies of *B. velezensis* Bv-116 cultured on skim milk agar and sodium carboxymethylcellulose solid media exhibited obvious clear zones around them, while no clear zones were observed when cultured on chitin agar and Poria cocos powder media (**Figures 3B-E**). It was shown that *B. velezensis* Bv-116 possessed protease and cellulase activities without chitinase and β-1,3-glucanase activities.

**Figure 4:**
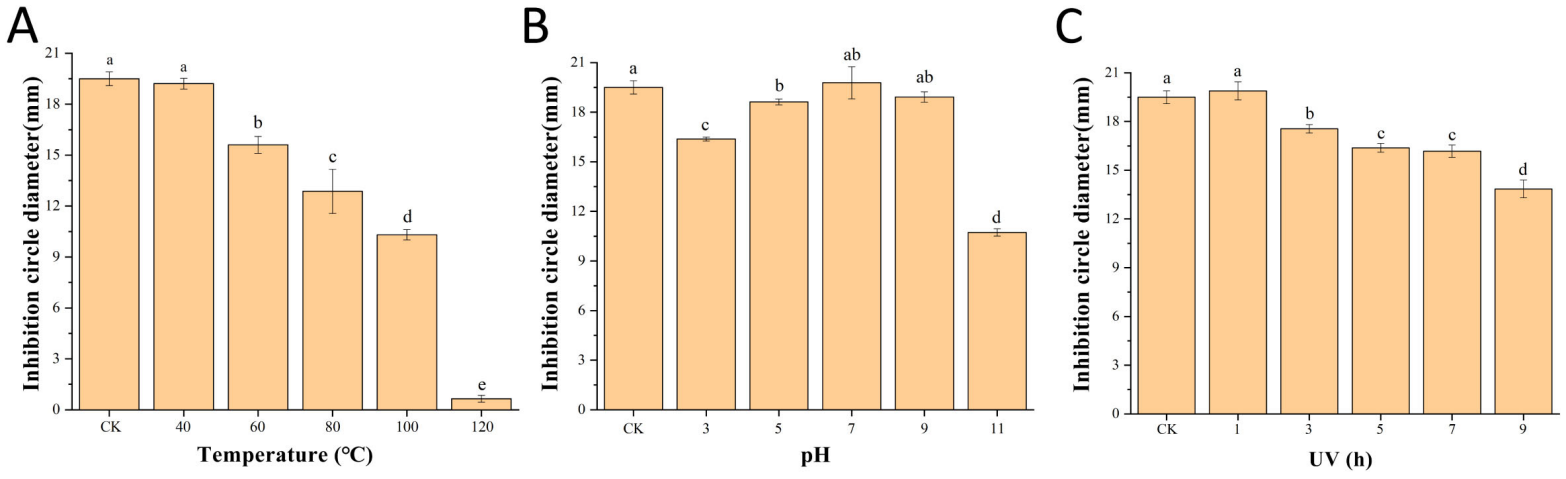
Antimicrobial stability of *B*. *velezensis* Bv-116 fermentation supernatant under different temperature **(A)**, pH **(B)** and UV **(C)** treatment. In the same picture, different letters (a-e) indicate significant differences at the *p* < 0.05 level.

The results of the antimicrobial stability test of the fermentation supernatant (**Figures 4A-C**) showed that the diameter of the inhibition circle remained at approximately 10.31 mm and the inhibitory activity was retained by 52.87% after treatment at 100°C for 15min. The pH of the original fermentation broth of *B. velezensis* Bv-116 was 7.2. The inhibitory activity remained well within the range of pH 3.0 to 9.0, and the diameter of the inhibition circle was still maintained at 10.73 mm with 55.03% inhibitory activity after pH 11.0 treatment for 12 h. After 9 h of UV irradiation, the diameter of the inhibition circle only decreased approximately 5.65 mm and the inhibitory activity was retained by 71.03%.

### 3.3 Study on the growth-promoting and biocontrol effects of *B. velezensis* Bv-116 and its bio-organic fertilizer

#### 3.3.1 Effect of *B. velezensis* Bv-116 and its bio-organic fertilizer on the growth promotion of cucumber seedlings

**Figure 5:**
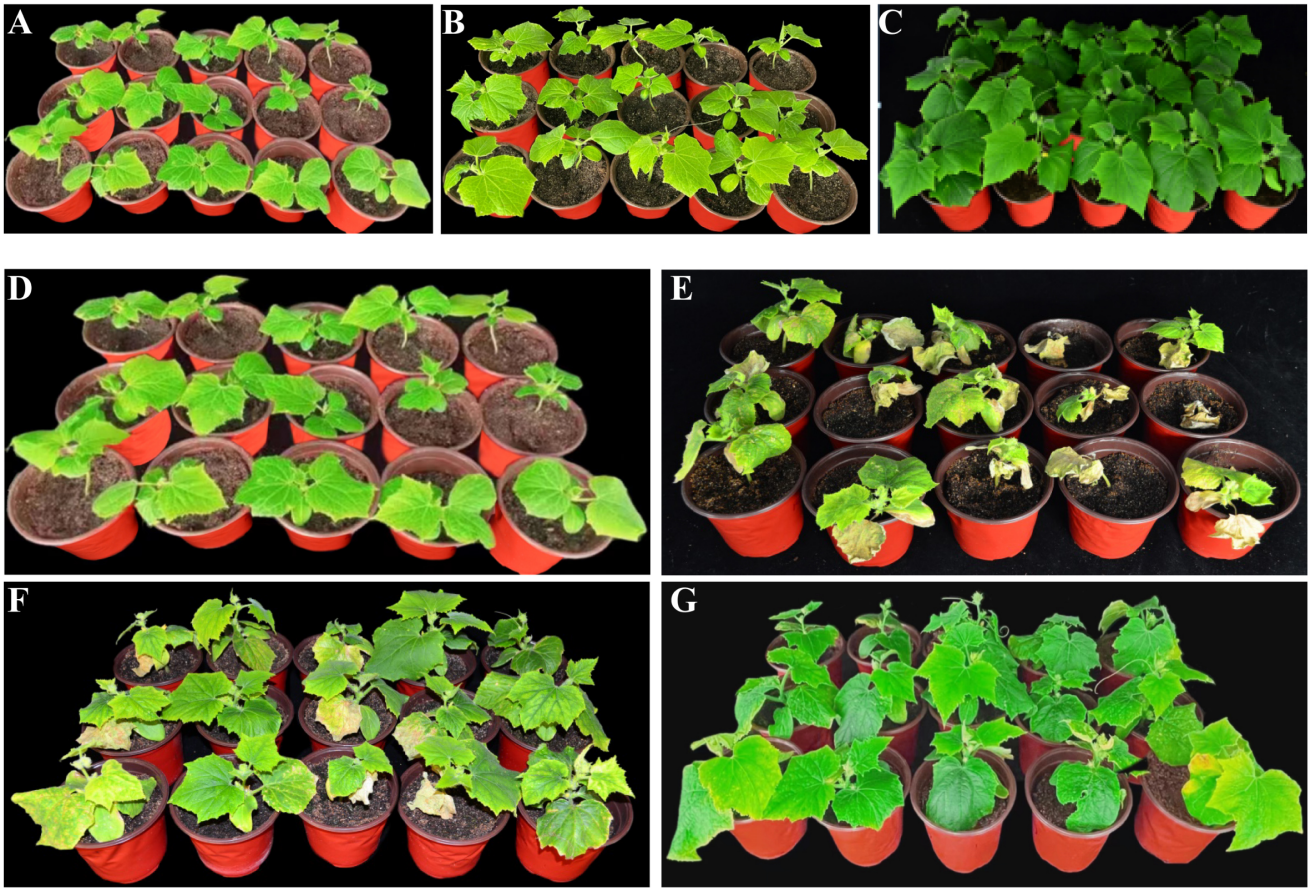
Growth promotion effect **(A–C)** of *B*. *velezensis* Bv-116 and its bio-organic fertilizer on cucumber seedlings and control effect **(D–G)** on cucumber *Fusarium* wilt disease. **(A)** is the control group; **(B)** is the treatment group inoculated with *B*. *velezensis* Bv-116; **(C)** is the group inoculated with bio-organic fertilizer; **(D)** is the control group; **(E)** is the FOC inoculation only treatment group; **(F)** is the FOC inoculation plus *B*. *velezensis* Bv-116 treatment group; **(G)** is the FOC inoculation plus bio-organic fertilizer treatment group.

The growth-promoting effects of *B. velezensis* Bv-116 and its bio-organic fertilizer on cucumber growth are illustrated in **Figures 5A-C**. Compared to the control group, significant increases in growth parameters of cucumber plants were observed after treatment with *B. velezensis* Bv-116 and its bio-organic fertilizer. Specifically, after 20 days of cultivation, cucumber plants treated with *B. velezensis* Bv-116 and its bio-organic fertilizer showed respective increases in plant height by 8.70% and 59.85%, stem diameter by 38.19% and 75.59%, leaf fresh weight by 148.87% and 482.64%, leaf dry weight by 137.78% and 520.00%, root fresh weight by 267.86% and 446.43%, and root dry weight by 266.67% and 700.00% (**Table 1**). In addition, there was no significant difference in chlorophyll a, chlorophyll b and chlorophyll a+b contents in Bv-116 group as compared to the control group, whereas the bio-organic fertilizer group showed increases of 63.55%, 50.98%, and 59.49%, respectively (**Table 2**).

**Table 1:** Growth parameters of cucumber in each treatment group after transplanting and cultivation of cucumber seedlings for 20 days.

Treatment group	Plant height (cm)	Stem diameter (mm)	(g per plant)			
			leaf fresh weight	Leaf dry weight	root fresh weight	root dry weight
CK	6.5 ± 0.14^a^	2.54 ± 0.04^c^	3.11 ± 0.28^a^	0.45 ± 0.02^c^	0.28 ± 0.05^a^	0.03 ± 0.00^c^
T1	7.1 ± 0.21^b^	3.51 ± 0.08^b^	7.74 ± 0.21^b^	1.07 ± 0.07^b^	1.03 ± 0.03^b^	0.11 ± 0.02^b^
T2	10.47 ± 0.17^c^	4.46 ± 0.04^a^	18.1 ± 0.17^c^	2.79 ± 0.04^a^	1.53 ± 0.06^c^	0.24 ± 0.06^a^

CK is the control group, T1 is the Bv-116 treatment group, and T2 is the bio-organic fertilizer treatment group. The data are presented as mean ± SD. Within the same column, different letters (a-c) indicate significant differences at p < 0.05 level.

**Table 2:** Chlorophyll content of cucumber leaves in each treatment group after transplanting and cultivation of cucumber seedlings for 20 days.

Treatment group	Chlorophyll a (mg/g)	Chlorophyll b (mg/g)	Chlorophyll a+b (mg/g)
CK	1.07 ± 0.04^a^	0.51 ± 0.02^b^	1.58 ± 0.05^a^
T1	1.03 ± 0.05^a^	0.53 ± 0.01^b^	1.56 ± 0.06^a^
T2	1.75 ± 0.03^b^	0.77 ± 0.02^a^	2.52 ± 0.05^b^

CK is the control group, T1 is the Bv-116 treatment group, and T2 is the bio-organic fertilizer treatment group. The data are presented as mean ± SD. Within the same column, different letters (a-b) indicate significant differences at p < 0.05 level.

#### 3.3.2 Control efficacy of *B. velezensis* Bv-116 and its bio-organic fertilizer on cucumber *Fusarium* wilt

After transplanting cucumber plants for 20 days, the growth status of cucumber plant indicated that FOC caused cucumber wilt. Meanwhile inoculation of *B. velezensis* Bv-116 and its bio-organic fertilizer exhibited effective inhibition against FOC-induced cucumber *Fusarium* wilt (**Figures 5D-G**). The disease incidence and disease severity index of cucumbers in the CK2 group reached 100% and 68.44, respectively. The disease incidence of cucumber plants in the T1 and T2 groups was 57.78% and 26.67%, respectively, representing reductions of 42.22% and 73.33% compared to the CK2 group. The disease severity index also decreased to 16.89 and 5.33, respectively, with control effects of 75.11% and 92.10%, respectively (**Supplementary Table S3**).

### 3.4 Analysis of cucumber rhizosphere soil microbial community

#### 3.4.1 Analysis of cucumber rhizosphere soil fungal community and their correlation with cucumber *Fusarium* wilt incidence

**Figure 6:**
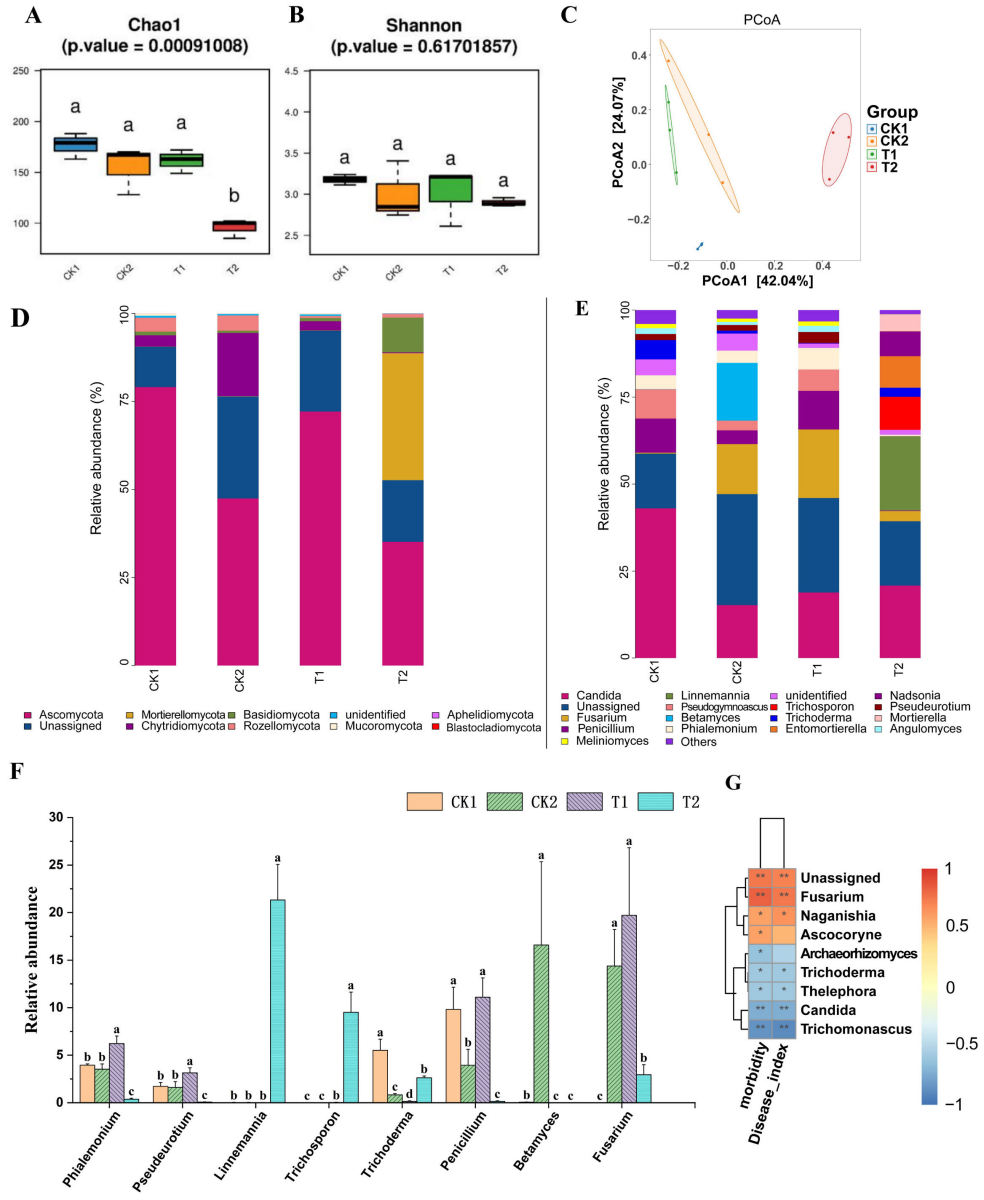
Analysis of cucumber rhizosphere soil fungal community and their correlation with cucumber *Fusarium* wilt incidence. **(A, B)** Analysis of the alpha diversity of soil fungal communities in cucumber rhizosphere soil; **(C)** PCoA of fungal communities based on beta diversity analysis; Composition of fungal community at phylum level **(D)** and genus level **(E)** in cucumber rhizosphere soil based on relative quantitative analysis; **(F)** The relative abundance of certain fungal genera in the rhizosphere soil of cucumber in each treatment group. **(G)** Correlation analysis of relative abundance of cucumber rhizosphere soil fungal communities at genus level with cucumber *Fusarium* wilt incidence and severity index based on Spearman’s correlation analysis. CK1 is the control group; CK2 is the treatment group with FOC inoculation; T1 is the treatment group with FOC and *B*. *velezensis* Bv-116 inoculation; T2 is the treatment group with FOC inoculation plus bio-organic fertilizer. In the same picture, different letters (a-d) indicate significant differences at the *p* < 0.05 level, * and ** indicate significant differences at the *p* < 0.05 level and *p* < 0.01 level, respectively.

The results of alpha diversity analysis of the fungal community in the rhizosphere soil of cucumber plants 20 days after transplanting (**Figures 6A, B**) showed that the Chao1 and Shannon indices of the T1 group, and the Shannon index of the T2 group, were not significantly different from those of the CK1 and CK2 groups. However, the Chao1 index of the T2 group was significantly lower than that of the CK1 and CK2 groups (*p* < 0.05). The results of principal coordinate analysis (PCoA) of fungal community, based on beta diversity analysis (**Figure 6C**), showed that there was no significant separation between the T1 group and the CK1 and CK2 groups. However, there was a significant separation between the T2 group and both the CK1, CK2, and T1 groups (*p* < 0.05).

The composition of fungal communities at the phylum level is depicted in **Figure 6D**. The phylum Ascomycota exhibited the highest abundance in groups CK1, CK2, and T1, whereas its abundance decreased in group T2. Abundance of the phylum Chytridiomycota was significantly higher in group CK2 compared to group CK1 (*p* < 0.05). In groups T1 and T2, abundance of both Chytridiomycota and Rozellomycota was significantly lower than that in group CK2 (*p* < 0.05), while abundance of Mortierellomycota and Basidiomycota in group T2 was significantly higher than that in groups CK2 and T1 (*p* < 0.05) (**Supplementary Figure S4**).

The composition of fungal communities at the genus level is illustrated in **Figure 6E**. Abundance of *Trichoderma* and *Penicillium* in group CK2 was significantly lower than that in the CK1 group, which received sterile water treatment, while abundance of *Betamyces* was significantly higher than that in the other three groups (*p* < 0.05). In group T1, abundance of *Phialemonium*, *Pseudeurotium*, and *Penicillium* was significantly higher than that in group CK2 (*p* < 0.05), with no significant difference observed in the abundance of *Fusarium* compared to group CK2. Moreover, in group T2, abundance of *Trichoderma*, *Linnemannia*, and *Trichosporon* was significantly higher than that in groups CK2 and T1, whereas abundance of *Fusarium* was significantly lower than that in groups CK2 and T1 (*p* < 0.05) (**Figure 6F**).

The results of Spearman’s correlation analysis, as illustrated in **Figure 6G**, elucidated the correlation between the relative abundance of soil fungal communities in the rhizosphere soil of cucumber plants at the genus level and the incidence as well as severity index of cucumber *Fusarium* wilt. The incidence and severity index of cucumber *Fusarium* wilt exhibited a significant positive correlation with the abundance of the genus *Fusarium*, to which the pathogen belongs (*p* < 0.05). Conversely, a significant negative correlation was observed between the abundance of the genus *Trichoderma*, which has a biocontrol role, and the incidence and severity index of cucumber *Fusarium* wilt (*p* < 0.05).

#### 3.4.2 Analysis of cucumber rhizosphere soil bacterial community and their correlation with cucumber *Fusarium* wilt incidence

**Figure 7:**
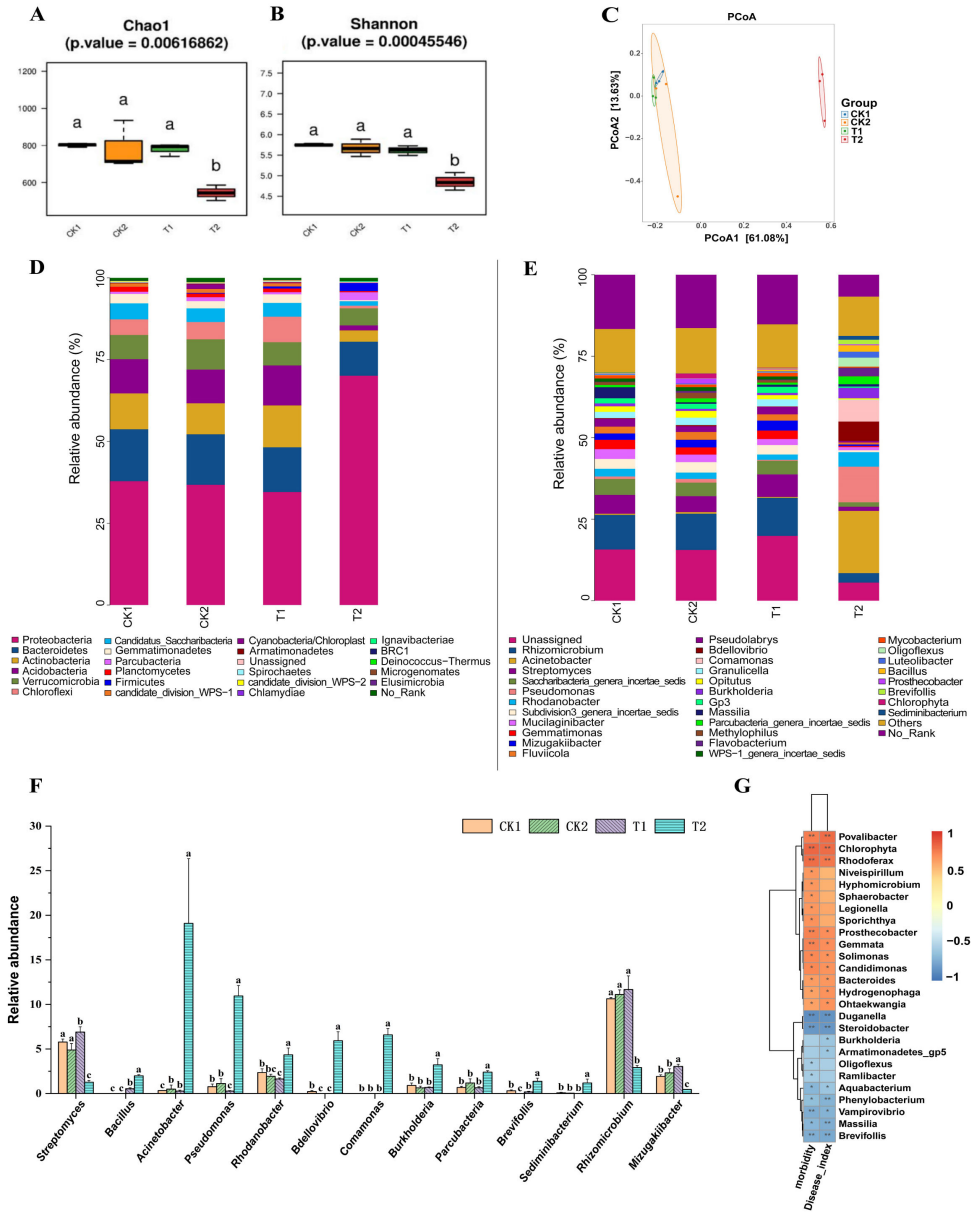
Analysis of cucumber rhizosphere soil bacterial community and their correlation with cucumber *Fusarium* wilt incidence. **(A, B)** Analysis of the alpha diversity of soil bacterial communities in cucumber rhizosphere soil; **(C)** PCoA of bacterial communities based on beta diversity analysis; Composition of bacterial community at phylum level **(D)** and genus level **(E)** in cucumber rhizosphere soil based on relative quantitative analysis; **(F)** The relative abundance of certain bacterial genera in the rhizosphere soil of cucumber in each treatment group. **(G)** Correlation analysis of relative abundance of cucumber rhizosphere soil bacterial communities at genus level with cucumber *Fusarium* wilt incidence and severity index based on Spearman’s correlation analysis. CK1 is the control group; CK2 is the treatment group with FOC inoculation; T1 is the treatment group with FOC and *B*. *velezensis* Bv-116 inoculation; T2 is the treatment group with FOC inoculation plus bio-organic fertilizer. In the same picture, different letters (a-c) indicate significant differences at the *p* < 0.05 level, * and ** indicate significant differences at the *p* < 0.05 level and *p* < 0.01 level, respectively.

The results of alpha diversity analysis of the bacterial community in the rhizosphere soil of cucumber plants 20 days after transplanting (**Figures 7A, B**) showed that the Chao1 and Shannon indices of the T1 group were not significantly different from those of the CK1 and CK2 groups, whereas both the Chao1 and Shannon indices of the T2 group were significantly lower than those of the CK1 and CK2 groups (*p* < 0.05). The results of PCoA of the bacterial community based on beta diversity analysis (**Figure 7C**) were similar to those of the fungal community, with no significant separation between the T1 group and the CK1 and CK2 groups, whereas there was a significant separation between the T2 group and both the CK1, CK2, and T1 groups (*p* < 0.05).

The composition of bacterial communities at the phylum level is depicted in **Figure 7D**. Proteobacteria, Bacteroidetes, Actinobacteria, Acidobacteria and Verrucomicrobia were the dominant bacteria in all treatment groups. Compared with other treatment groups, abundance of Proteobacteria in group T2 was significantly increased (*p* < 0.05). At the same time, abundance of Bacteroidetes, Actinobacteria, Acidobacteria and Ver-rucomicrobia was significantly decreased (*p* < 0.05) (**Supplementary Figure S5**).

The composition of bacterial communities at the genus level is illustrated in **Figure 7E**. Compared with CK1 group, abundance of *Acinetobacter* in CK2 group was significantly increased (*p* < 0.05), while abundance of *Bdellovibrio* and *Brevifollis* was significantly decreased (*p* < 0.05). Abundance of *Bacillus*, *Streptomyces* and *Mizugakiibacter* in T1 group was significantly higher than that in CK2 group (*p* < 0.05), while the abundance of *Pseudomonas* in T1 group was significantly lower than that in CK2 group (*p* < 0.05). Abundance of *Bacillus*, *Pseudomonas*, *Burkholderia*, *Brevifollis, Parcubacteria*, *Comamonas*, *Sediminibacterium* and *Rhodanobacter* in T2 group was significantly higher than that in CK2 and T1 groups (*p* < 0.05). Abundance of *Streptomyces*, *Rhizomicrobium* and *Mizugakiibacter* was significantly lower than that of CK2 and T1 groups (*p* < 0.05) (**Figure 7F**).

The results of Spearman’s correlation analysis, as illustrated in **Figure 7G**, elucidated the correlation between the relative abundance of soil bacterial communities in the rhizosphere soil of cucumber plants at the genus level and the incidence as well as severity index of cucumber *Fusarium* wilt. Cucumber wilt incidence and disease severity index were significantly and positively correlated with the abundance of the genera *Povalibacter*, *Chlorophyta*, and *Rhodoferax* (*p* < 0.01), and significantly and negatively correlated with the abundance of the genera *Brevifollis*, *Steroidobacter* and *Duganella* (*p* < 0.01). In addition, abundance of *Burkholderia*, an important class of biocontrol bacteria (Lahlali et al., 2022), was significantly (*p* < 0.05) negatively correlated with the cucumber *Fusarium* wilt disease severity index.

## 4 Discussion

Cucumber *Fusarium* wilt caused by FOC is one of the main causes of cucumber yield reduction (Bartholomew et al., 2022). The use of biocontrol strains for wilt control has become a current research hotspot as an environmentally friendly and effective strategy (Sarrocco, 2023).

As a biocontrol bacterium characterized by broad-spectrum antimicrobial activity, strong resistance to adversity, diverse biological control mechanisms, environmental friendliness, and safety to humans and animals, *B. velezensis* has gained increasing attention (Yang et al., 2023). In this study, *B. velezensis* Bv-116 was screened out from 565 bacteria strains preserved in the laboratory, which exhibited significant inhibitory effects against FOC. Furthermore, *B. velezensis* Bv-116 showed strong antagonistic activity against five tested fungi, including *Fusarium graminearum*, indicating its broad-spectrum antifungal potential. In pot experiments, *B. velezensis* Bv-116 and its bio-organic fertilizer treatment showed disease control efficiencies of 75.11% and 92.10% against cucumber *Fusarium* wilt, respectively, indicating its potential as biocontrol agents against cucumber *Fusarium* wilt.

Research has shown that certain *bacillus* strains can achieve disease control by inhibiting the growth of pathogens through the production of lipopeptide antibiotics (Shen et al., 2023), volatiles (Ling et al., 2022), extracellular enzymes (Li et al., 2021), inducing plant resistance (Tahir et al., 2017), and competing for nutrients and ecological niches with the pathogens (Zhang et al., 2020a). Specifically, Ling et al. (2022) revealed significant preventive effects of volatile organic compounds (VOCs) released by *B. velezensis* L1 on the pathogenic fungi causing Lycium fruit rot. *B. velezensis* Mr12 exhibits broad-spectrum antagonism against various plant pathogens, with high stability of inhibitory substances and the ability to produce various peptide polysaccharides and polyketides as well as cell wall hydrolases (Li et al., 2021). Our research results indicate that *B. velezensis* Bv-116 possesses strong protease and cellulase activities. Its fermentation supernatant significantly inhibits the spore germination of FOC, disrupts hyphal morphology, and affects the relative permeability of FOC cell membranes. It is speculated that *B. velezensis* Bv-116 may degrade the cell wall of FOC by secreting proteases and cellulases, disrupting the hyphal cell membrane, causing it to lose its original function, leading to leakage of cell contents, thereby achieving the purpose of inhibiting or killing FOC. When the inhibited mycelia of FOC are recultured, their growth activity significantly decreases, and their growth rate becomes slow. This may be related to the damage to the mycelial cell structure, requiring time for self-repair.

In addition, we found that the volatile gases produced by *B. velezensis* Bv-116 inhibited the mycelial growth of FOC, which may also play an important role in controlling soil-borne diseases caused by FOC. And the components of antifungal activity in its volatile organic compounds (VOCs) still need to be further investigated. VOCs produced by different biocontrol bacteria act in different ways, such as inhibiting fungal mycelial growth (Ling et al., 2022), inducing plant systemic resistance (Tahir et al., 2017), and promoting plant growth (Gao et al., 2022). The fermentation supernatant of *B. velezensis* Bv-116 has good antimicrobial stability, which is similar to other reported *bacillus* (Jia et al., 2018; Huang et al., 2022). For example, The bacteriostatic effect and stability of *Bacillus velezensis* JK19 fermentation supernatant under different temperatures, pH, enzyme treatment and ultraviolet irradiation were determined by TTC double-layer plate confrontation method, and the results showed that strain JK19 had strong antibacterial stability and antibacterial activity (Huang et al., 2022); the fermentation supernatants of *Bacillus thuringiensis* (BT)Bt185 and HD-1 were stable to heat, acid, ultraviolet radiation and continuous ultrasonic stimulation (Jia et al., 2018). This indicates its potential use as a biocontrol agent or in the production of bio-organic fertilizer. Subsequent research requires whole-genome sequencing and analysis of *B. velezensis* Bv-116 to further explore its potential biocontrol functions and mechanisms of action.

The beneficial microorganisms can promote plant growth and biomass accumulation (Han et al., 2014). This is consistent with our research findings, namely that the application of *B. velezensis* Bv-116 and its bio-organic fertilizer significantly promotes the growth of cucumbers. *B. velezensis* was reported to produce indole-3-acetic acid, thereby promoting the growth of cucumber plants (Lobo et al., 2023). It is speculated that the growth-promoting effect of *B. velezensis* Bv-116 may be related to this. Additionally, studies have shown that combining antagonistic strains with organic fertilizer to prepare bio-organic fertilizer can serve as high-quality carriers for antagonistic strains while providing high-quality nutrients for plants (Zhang et al., 2020b). In this study, *B. velezensis* Bv-116 was added to kitchen waste to prepare bio-organic fertilizer. This not only helps conserve resources and reduce the secondary pollution caused by landfilling and incineration of waste but also kitchen waste can provide rich nutrients for plants after fermentation. This may be one of the potential reasons why the growth indicators of cucumber, the chlorophyll content in leaves, and the control effect on cucumber *Fusarium* wilt in the Bv-116 bio-organic fertilizer treatment group are higher than those in the other three groups.

On the other hand, rhizosphere microbial communities play an important role in promoting plant growth and increasing tolerance to diseases and abiotic stresses (Trivedi et al., 2021). In our study, it was found that the application of *B. velezensis* Bv-116 and its bio-organic fertilizer could change the microbial community structure in the rhizosphere soil of cucumber. In the potting disease prevention experiment, abundance of *Bacillus* genus in the T1 group with the addition of *B. velezensis* Bv-116 (0.54%) was significantly higher than that in the CK2 group (< 0.01%), demonstrating a better disease control effect. It is speculated that the addition of *B. velezensis* Bv-116 may increase the abundance of *Bacillus*, *Penicillium* (Marfetan et al., 2023), *Streptomyces* (Dow et al., 2023), and other genera with biological control effects on various plant diseases. The disease control effect of the T2 group supplemented with bioorganic fertilizer was superior to that of the T1 group. This may be due to a significant increase in the abundance of genera possessing biocontrol functions against various plant diseases in the T2 group, including *Bacillus* associated with *B. velezensis* Bv-116, *Pseudomonas* (Yan et al., 2013), *Burkholderia* (Lahlali et al., 2022) and *Trichoderma* (Guzmán-Guzmán et al., 2023), etc., compared to the T1 group. While abundance of *Fusarium* was significantly reduced in T2 group compared to the T1 group. It has been reported that ferulic acid, p-hydroxybenzoic acid and coumaric acid produced from the decomposition of kitchen waste organic materials can also directly inhibit the growth of pathogens, thus reducing the attack of cucumber *Fusarium* wilt (Zaman and Purwono, 2022). At the same time, the growth-promoting effect of *B. velezensis* Bv-116 and its bio-organic fertilizer on cucumber plants may also enhance the disease resistance of cucumber plants themselves. In addition, compared to both the CK2 and T1 groups, the addition of bioorganic fertilizer resulted in a significantly higher abundance of genera such as *Comamonas*, *Sediminibacterium*, and *Rhodanobacter* in the T2 group, which possess the capability to degrade heavy metals, pesticides, and other environmental pollutants (Zhao et al., 2012; Fang et al., 2024), conversely, phyla associated with soil infertility, such as Acidobacteria and Verrucomicrobia (Finn et al., 2017), exhibited significantly lower abundance in the T2 groups.

In conclusion, *B. velezensis* Bv-116 exhibits the capability to inhibit FOC growth by disrupting its cell structure and generating volatile antimicrobial gases. In the presence of pathogens, the addition of *B. velezensis* Bv-116 or its bio-organic fertilizer prepared by adding it to kitchen waste and fermenting it to cucumber transplantation soil increased the abundance of *Bacillus* in the rhizosphere soil of cucumber, promoted the growth of cucumber, and had a biocontrol effect on cucumber *Fusarium* wilt. The application of *B. velezensis* Bv-116, a functional strain antagonistic to FOC, and the bio-organic fertilizer prepared by adding it to kitchen waste and fermenting it may be a potential environmentally friendly biocontrol strategy against cucumber *Fusarium* wilt.

## Data Availability

The datasets presented in this study can be found in online repositories. The names of the repository/repositories and accession number(s) can be found in the article/**Supplementary Material**. This data can be found here: https://ngdc.cncb.ac.cn/bioproject/browse/; PRJNA1118050.
