# Supplementary material for: Inoculation of Bacillus velezensis Bv-116 and its bio-organic fertilizer serve as an environmental friendly biocontrol strategy against cucumber Fusarium wilt
Source: Front Plant Sci. 2024 Oct 15;15:1467265. doi: 10.3389/fpls.2024.1467265 (PMC11518806; doi:10.3389/fpls.2024.1467265)
Supplement: Supplementary file 1 [file DataSheet1.docx]

**Table S1.** Antagonistic activity of the 10 strains against FOC.

| Antagonistic bacteria | Pathogen colony diameter (mm) | inhibition rate  (%) |
| --- | --- | --- |
| Bv-116 | 16.21±0.22 ^f^ | 84.93±0.14 ^a^ |
| Ba-024 | 18.25±0.34 ^e^ | 81.25±0.11 ^b^ |
| Ef-057 | 18.47±0.16 ^e^ | 80.88±0.56 ^b^ |
| Ba-201 | 20.66±0.38 ^d^ | 76.84±0.22 ^c^ |
| Bv-338 | 20.85±0.43 ^d^ | 76.47±0.27 ^c^ |
| Bs-157 | 21.44±0.48 ^c^ | 75.37±0.55 ^d^ |
| Bs-203 | 24.24±0.24 ^b^ | 70.22±0.14 ^e^ |
| Ba-441 | 24.88±0.52 ^b^ | 69.12±0.66 ^e^ |
| Ba-177 | 25.04±0.38 ^a^ | 68.75±0.31 ^f^ |
| Bs-266 | 25.43±0.11 ^a^ | 68.01±0.15 ^f^ |

The data are presented as mean ± standard deviation (SD). Within the same column, different letters (a-f) indicate significant differences at *p* < 0.05 level.

**Table S2.** Inhibition rate of *B. velezensis* Bv-116 against five fungi

| Fungi | Fungal colony diameter (mm) | Inhibition rate (%) |
| --- | --- | --- |
| *Trichoderma longibrachiatum* | 11.51±0.32^e^ | 94.00±014^a^ |
| *Aspergillus niger* | 8.50±0.20^d^ | 97.60±0.27^b^ |
| *Aspergillus oryzae* | 9.14±0.33^c^ | 93.79±0.52^c^ |
| *Aspergillus flavus* | 9.07±0.50^b^ | 94.22±0.25^d^ |
| *Fusarium graminearum* | 16.17±0.17^a^ | 87.68±0.17^e^ |

The data are presented as mean ± SD. Within the same column, different letters (a-e) indicate signifcant differences at *p* < 0.05 level.

**Table S3.** Effect of *B. velezensis* Bv-116 and its bio-organic fertilizers on the control of cucumber *Fusarium* wilt

| Treatment group | DI (%) | DSI | CE (%) |
| --- | --- | --- | --- |
| CK1 | 0.00±0.00^d^ | 0.00±0.00^d^ | - |
| CK2 | 100.00±0.00^a^ | 68.44±4.68^a^ | - |
| T1 | 57.78±3.85^b^ | 16.89±2.04^b^ | 75.11±4.61^b^ |
| T2 | 26.67±6.67^c^ | 5.33±1.33^c^ | 92.10±2.48^a^ |

CK1 is the control group; CK2 is the FOC inoculation only treatment group; T1 is the FOC inoculation plus *B. velezensis* Bv-116 treatment group; T2 is the FOC inoculation plus bio-organic fertilizer treatment group. The data are presented as mean ± SD. Within the same column, different letters (a-d) indicate significant differences at p < 0.05 level.

**
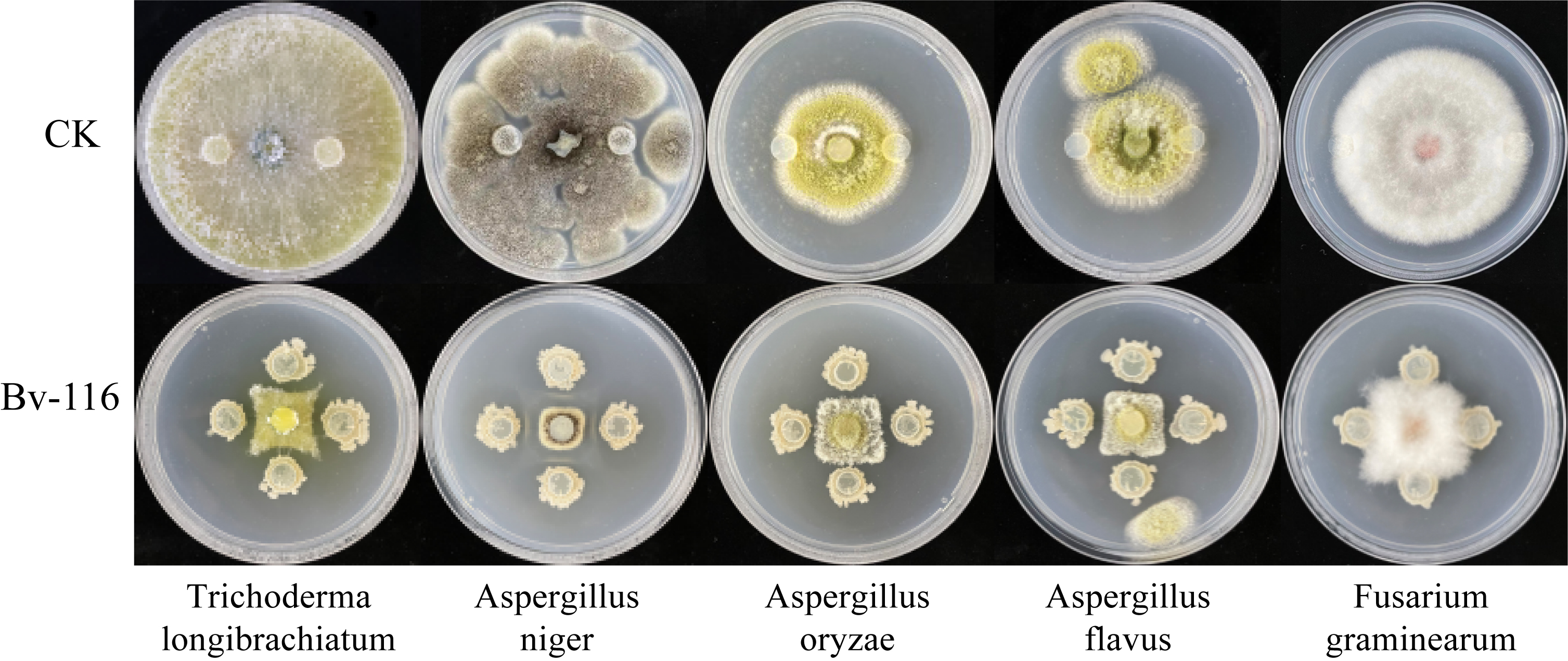
**

**Figure S1.** Antagonistic activity of *B. velezensis* Bv-116 against five fungi.

**Figure S2.** Inhibition rate of spore germination of FOC with different concentration of *B. velezensis* Bv-116 fermentation supernatant. In the same picture, different letters (a-c) indicate significant differences at the *p* < 0.05 level.


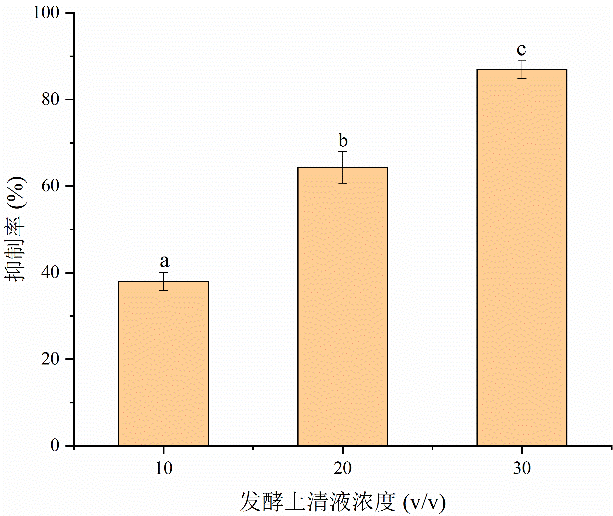

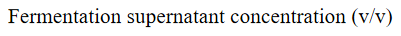

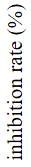


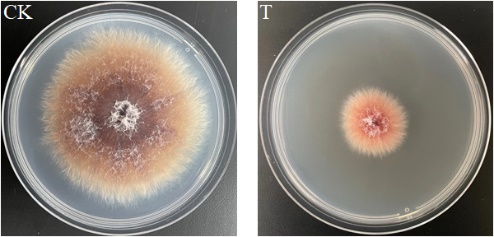


**Figure S3.** The growth of inhibited mycelium reculture. CK is the control group; T is the treatment group


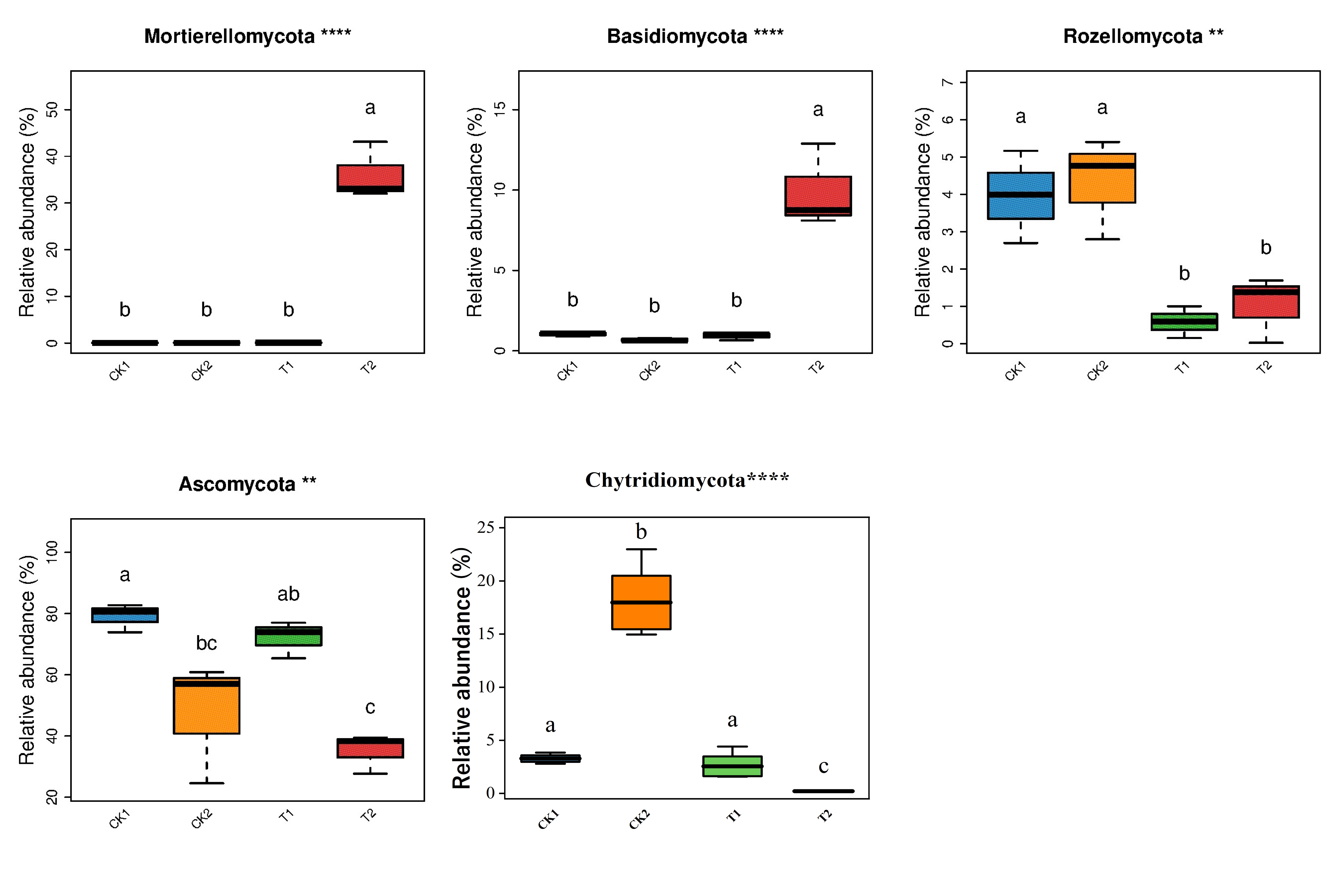


**Figure S4.** The relative abundance of certain fungal phyla in the rhizosphere soil of cucumber in each treatment group. In the same picture, different letters (a-c) indicate significant differences, ** and **** indicate significant differences at the *p* < 0.01 level and *p* < 0.0001 level, respectively.


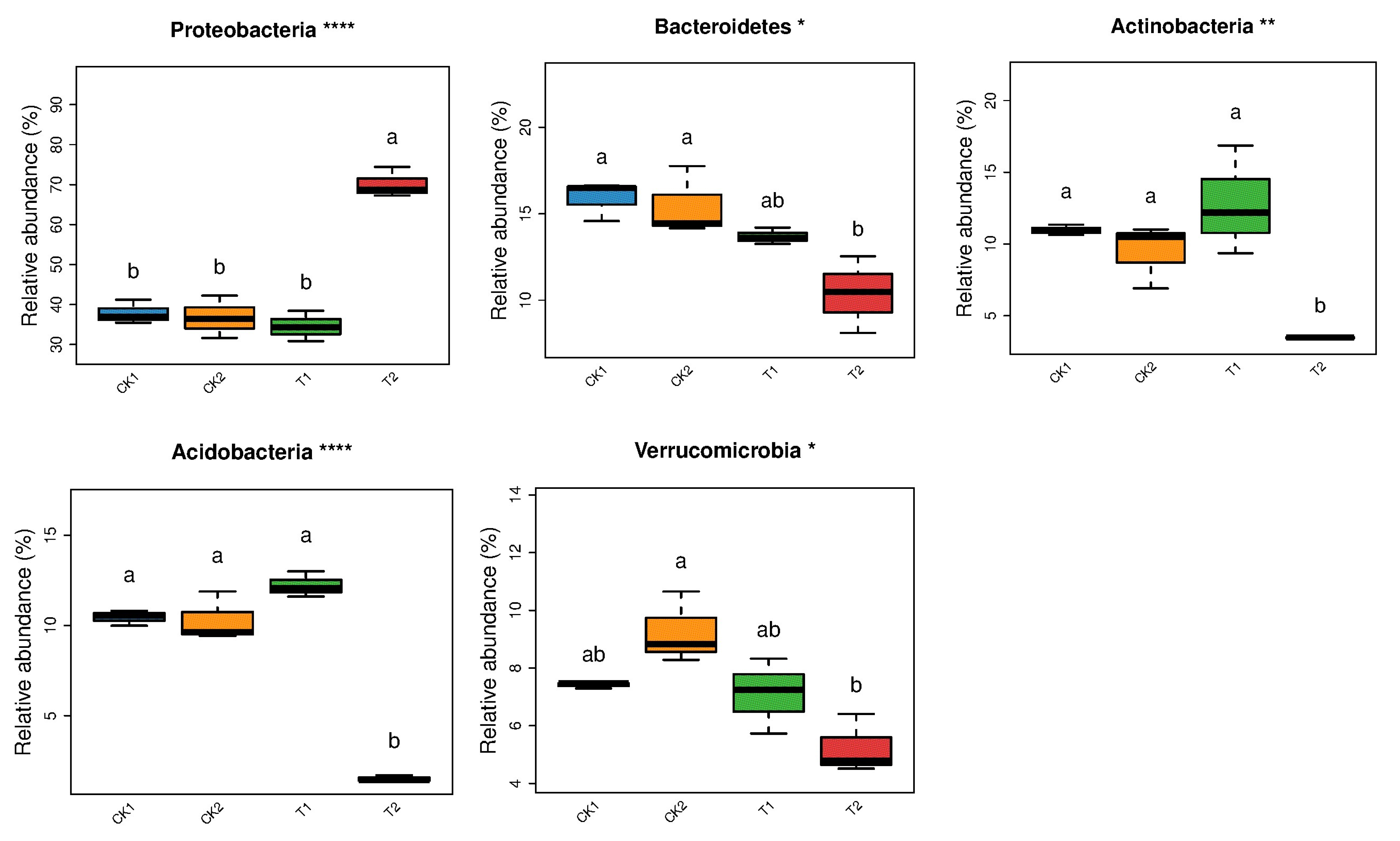
**Figure S5.** The relative abundance of certain bacterial phyla in the rhizosphere soil of cucumber in each treatment group. In the same picture, different letters (a-c) indicate significant differences, *, ** and **** indicate significant differences at the *p* < 0.05, *p* < 0.01 and *p* < 0.0001 level, respectively.
